# The effects of measurement errors on minimum ablative margins after thermal ablation of liver tumors: a simulation study

**DOI:** 10.1007/s44343-025-00029-9

**Published:** 2026-01-14

**Authors:** Iwan Paolucci, Jessica Albuquerque, Noreen S. Siddiqi, A. Kyle Jones, Kristy K. Brock, Bruno C. Odisio

**Affiliations:** 1 Department of Interventional Radiology, The University of Texas MD Anderson Cancer Center, Houston, TX USA; 2 Department of Imaging Physics, The University of Texas MD Anderson Cancer Center, Houston, TX USA

**Keywords:** Liver malignancies, Thermal ablation, Minimum ablative margin, Simulation

## Abstract

**Purpose:**

To develop a mathematical framework to estimate the in silico A0 threshold based on the technical specifications of a specific ablation confirmation software package for thermal ablation of liver tumors that can then be used to identify the impact of different sources of error.

**Methods:**

To estimate in silico A0 thresholds, we developed a simulation framework incorporating technical parameters and biological effects. Technical parameters were segmentation error, registration error, and slice thickness, and biological effects were tissue shrinkage and microscopic satellite lesions; these parameters and effects were all modeled using statistical distributions. For each permutation of parameters, a logistic regression was fitted to determine the observed MAM required to achieve ≥ 99% probability of true complete tumor coverage (i.e., the A0 threshold). The mathematical framework was integrated into a web application to estimate the A0 threshold and the reliability of the commonly used 5-mm A0 threshold based on several software performance characteristics.

**Results:**

A total of 15,000,000 simulations (10,000 simulations × 1500 parameter permutations) were run and summarized. Tumor and ablation zone segmentation most greatly influenced the A0 threshold, with thresholds of 3.4 and 8.4 mm for 1- and 5-mm errors, whereas slice thickness had a relatively small effect, with A0 thresholds of 2.9 and 3.4 mm for thicknesses of 1 and 5 mm, respectively.

**Conclusion:**

This framework provides a method to determine software-specific in silico A0 thresholds and evaluate the reliability of existing 5-mm criteria based on software performance metrics. The results further show that ablation confirmation software should have registration and segmentation errors of ≤ 3 mm to reliably use a 5-mm A0 threshold.

## Introduction

Thermal ablation is a minimally invasive local curative-intent treatment option for patients with small (≤ 3–5 cm) primary and secondary liver tumors [1]. It is part of national guidelines for hepatocellular carcinomas, intrahepatic cholangiocarcinoma, and colorectal liver metastases [2–6] and also frequently used in other metastatic settings. Over the past decade, image guidance for thermal ablation has drastically improved with the introduction of stereotactic guidance, robotics, and software-based ablation confirmation. Software-based ablation confirmation is used to ensure that the minimum ablative margin (MAM), which has been repeatedly shown to be the most important predictor of local tumor progression [7–10], is achieved during the procedure.

Similar to R0 resection, A0 ablation has been defined as ablation with complete tumor coverage that includes a pre-specified margin (e.g., MAM ≥ 5 mm) and a low risk of local tumor progression [11]. However, the true MAM cannot be measured microscopically or histologically because the tumor is not surgically removed; instead, it is destroyed by the ablation in situ and only necrosis remains within the liver. Thus, MAM is an imaging-based surrogate of histopathologic margin measured by co-registering the pre-ablation and post-ablation images. A recent systematic review reinforced 5 mm as a minimum requirement but stated 10 mm as optimal outcomes [12]. The study also identified high heterogeneity among studies which could be due to significant differences in measurement methodologies and their accuracies. For quantitative and objective measurements, segmentation methods are used to contour the tumor and ablation zone and compute the MAM in all 3 dimensions. Because this image processing is time consuming, automated algorithms based on convolutional neural networks have been developed and integrated into dedicated MAM confirmation software (also called ablation confirmation [AC] software) packages [13, 14].

Many AC software platforms lack robust clinical evidence establishing a clear and reproducible A0 margin threshold within the measurement accuracy of their respective systems. Specific thresholds for each AC software are often determined in retrospective series. Previously published and ongoing trials often use a MAM of 5 or 10 mm as sufficient (A0) margins [15–18]. However, newer studies using more advanced and accurate imaging processing techniques have identified lower thresholds [7]—indicating the influence of measurement accuracy on the A0 threshold. Conducting a clinical study to first find and then validate A0 in each software package is impractical, as these packages evolve and provide newer and more accurate image processing methods regularly. Thus, in silico methods for initial technical validation would be a valuable tool.

In this study, we present a mathematical framework to estimate the in silico A0 threshold based on the technical specifications of the specific AC software package being used. The primary aim was to estimate the effect of registration and segmentation inaccuracies, as well as the slice thickness, on the threshold of A0 margins to further guide efforts to improve these technologies. The secondary aim was to use those estimates, to identify the minimum accuracy requirements needed to reliably use a 5-mm MAM threshold.

## Materials and methods

A recent review article identified 5 key sources of inaccuracy affecting A0 thresholds: image resolution, segmentation error, registration error, deformation, and image artifacts [19]. The specific parameters used in the simulation are presented in Table 1. For this study, the effects of image artifacts were considered part of segmentation error, and deformation was considered part of tissue shrinkage and registration error and not explicitly modeled. Further, for image resolution, only the slice thickness was included in this study since the in-plane resolution is typically < 1 mm.

**Table 1:** Parameter sets used in this study

Parameter	Notation	Values
Tissue shrinkage, %	${\varphi }_{\mathrm{Shrinkage}}$	0, 10, 20, 30
Microscopic satellite lesions, %	${p}_{\mathrm{Satellite}}$	0, 25, 50, 75, 100
Registration error, mm	${\varepsilon }_{\mathrm{Registration}}$	1, 2, 3, 4, 5
Segmentation error, mm	${\varepsilon }_{\mathrm{Segmentation}}$	1, 2, 3, 4, 5
Slice thickness, mm	${\varphi }_{\text{Slice thickness}}$	1, 2, 3, 4, 5
Tumor size, mm	${d}_{\text{Tumor size}}$	10 – 30
Minimum ablative margin, mm	$MA{M}_{\mathrm{true}}$	−5 – 10

### Biological effects

**Fig. 1:**
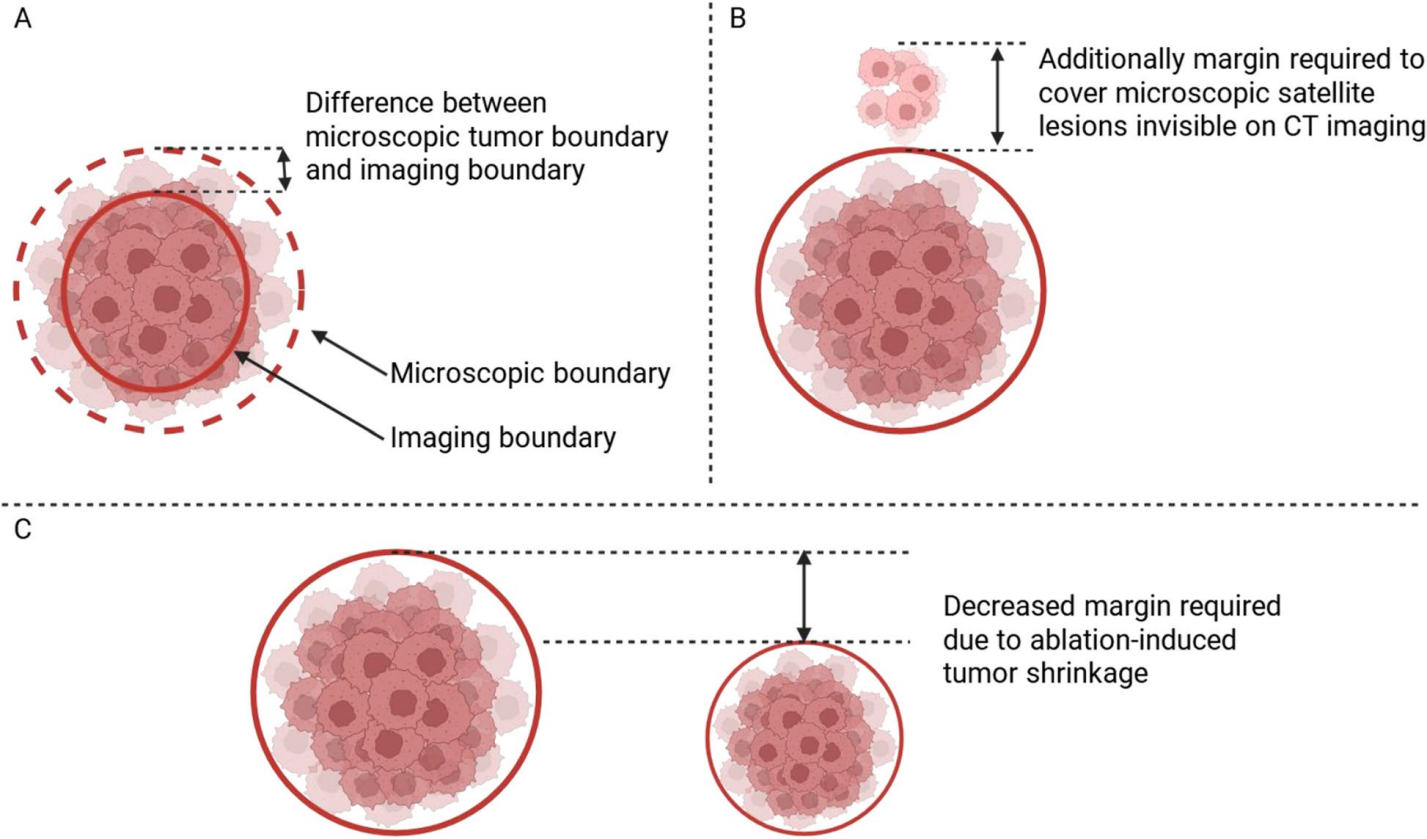
The effects of visibility of microscopic tumor on cross-sectional imaging (**A**), microscopic satellite lesions (**B**), and tissue shrinkage (**C**) on the required margins

We examined 2 major biological effects on MAM measurement: (i) tissue shrinkage due to the application of microwave ablation energy and (ii) the presence of microscopic satellite lesions not visible on imaging. Both effects influence the accuracy of the depiction of the actual tumor and ablation zone (Fig. 1).

#### Tissue shrinkage

Tissue shrinkage during microwave ablation has been studied mainly in ex vivo experiments, as in vivo studies involving patients undergoing microwave ablation are unfeasible. Radial tissue contraction has been reported to be up to 40% in ex vivo studies in bovine tissue [20]. This finding likely represents an upper limit because ex vivo bovine tissue is non-perfused and therefore dehydrates faster than perfused tissue. Tissue contraction within the ablation zone can cause the MAM to be overestimated. In addition, tumor tissue contraction cannot be observed during the procedure, and thus, the exact tumor-specific shrinkage is unknown. In our model, we assumed that the tissue shrinkage is normally distributed.

$${e}_{\mathrm{Shrinkage}}\sim \mathrm{Normal}\left(x, 0.05\right)$$

#### Microscopic satellite lesions

The accuracy of the measurement relies on the accurate depiction of microscopic disease on macroscopic imaging, which has an average spatial resolution of approximately 1 mm. Therefore, microscopic satellite lesions adjacent to the ablated tumor may be present but not visible on cross-sectional imaging. In addition, the transition zone between microscopic tumor and healthy tissue might not be accurately depicted on imaging, and therefore, the tumor size might be underestimated, resulting in an overestimated MAM. In our model, we assumed that microscopic satellite lesions are directly adjacent, the sizes are uniformly distributed between 0.5 and 2.5 mm, and the probability of the presence of microscopic satellite lesions follows a binomial distribution.

$${p}_{\mathrm{Satellite}} \sim \mathrm{Binomial}\left(1, p\right)$$

$${d}_{\mathrm{Satellite}}=\left\{\begin{array}{c}0, {p}_{\mathrm{Satellite}}=0\\ Uniform(0.5, 2.5), {p}_{\mathrm{Satellite}}=1\end{array}\right.$$

### Technical parameters

**Fig. 2:**
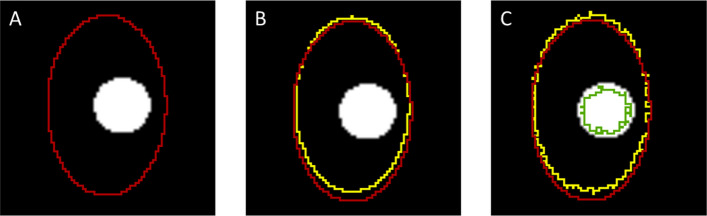
Intermediate results from an example case showing the true margin (**A**), application of the registration error (**B**), and application of random noise to the segmentation masks (**C**). The tumor is shown in white, the tumor contour in green, the true ablation zone in red, and the ablation zone influenced by measurement error in yellow

We examined the following sources of technical error: image registration, image segmentation variation, and slice thickness (Fig 2). These errors directly affect the accuracy of the MAM.

#### Registration error

Registration error significantly affects the accuracy of MAM measurements and is inherent to each specific registration method. In addition, it is often not possible to improve the registration accuracy during an ablation procedure with a given set of images. In our model, the registration error consists of a magnitude and a direction. The magnitude is normally distributed with a mean of 0 with varying standard deviation. The direction is chosen randomly in either *X*, *Y*, or *Z* direction, with each axis having the same probability of being chosen.

$${e}_{\mathrm{mag}}\sim \mathrm{Normal}\left(0, {e}_{\mathrm{Registration}}\right)$$

$${e}_{\mathrm{dir}}\in \left[X, Y, Z\right]$$

$${e}_{\mathrm{Shift}}= {e}_{\mathrm{dir}}\times {e}_{\mathrm{mag}}$$

The shift (${e}_{\mathrm{shift}}$) is then applied to the ablation center, whereas the tumor center remains constant.

#### Segmentation error

Segmentation of tumors and ablation zones varies between observers and can also be influenced by the available segmentation tools and artificial intelligence–based automated algorithms. In contrast to registration error, segmentation error can be minimized by having an experienced radiologist review artificial intelligence–based segmentation results and make necessary adjustments using manual tools such as brushes. However, for intra-procedural MAM assessment, the time to make such adjustments is limited, increasing the risk for segmentation errors. Segmentation errors are typically calculated using the Dice similarity coefficient, measuring the overlap of the contours, or the Hausdorff distance, measuring the distance between the contours [21, 22]. For our model, the segmentation error was estimated to be randomly distributed and added as random noise to the segmentation mask. In addition, the segmentation errors for the tumor and the ablation zone were assumed to be independent.

$${e}_{\mathrm{Tumor}}\sim \mathrm{Normal}\left(0, 1\right)$$

$${e}_{\mathrm{Ablation}}\sim \mathrm{Normal}(0, 1)$$

#### Slice thickness

Recent studies on AC software have used slice thicknesses between 1 and 5 mm [23–25]. The slice thickness interacts with the segmentation and registration process because thin slices typically result in a lower signal-to-noise ratio, reducing tumor depiction. Slice thickness also limits the precision of the measurement in the cranio-caudal direction. In addition, thinner slices result in more slices and thus a higher computational cost and more manual work. In our model, the interactions of slice thickness with other errors and computing time were ignored. To analyze the effect of slice thickness, the tumor and ablation segmentation mask were reconstructed with an in-plane resolution of 1 mm but varying resolution in cranio-caudal direction denoted as *φ*_Slice thickness_.

### Simulations

#### Approach and parameters

For simulations, we considered a set of parameters for each influencing factor (Table 1). The data were processed as follows in each simulation:

1. Randomly sample tumor size and ablative margin
2. Create 2 images with a tumor and ablation zone based on the size drawn in step 1
3. Apply tissue shrinkage and microscopic satellite lesions to tumor
4. Measure true MAM (*MAM*_*True*_)
5. Apply random registration error to ablation zone
6. Apply random noise to the tumor and ablation segmentation masks
7. Resample both images with given slice thickness
8. Measure observed MAM (*MAM*_*Observed*_)

For each permutation of parameters (*N* = 1500), 10,000 simulations were performed, and the results were recorded. The simulations were implemented in Python (version 3.12). Synthetic image generation and processing were implemented using NiBabel (version 5.2.0) and NumPy (version 1.24.4). The “qam” package was used to quantify the minimum ablative margins [26].

#### Estimating A0 and probability of complete coverage

We defined the A0 threshold as the observed MAM where the true MAM is > 0 (complete microscopic tumor coverage) at least 99% of the time. Mathematically, this is denoted as:

$$P\left(MA{M}_{\mathrm{true}}>0 \right| MA{M}_{\mathrm{observed}}>{x}_{\mathrm{A}0})\ge 99\%$$

To calculate the A0 threshold, we fitted a logistic regression model to estimate the sigmoid function on a continuous scale of the observed MAM from − 5 mm to 10 mm. We then located the observed MAM where the probability of true MAM is greater than 99% and defined it as A0. In addition, we estimated the probability of complete coverage (> 0-mm *MAM*_true_) if the commonly used 5-mm MAM threshold is observed as follows:

$$P\left(MA{M}_{\mathrm{true}}>0 \right| MA{M}_{\mathrm{observed}}\ge 5)$$

### Web application for A0 and 5-mm MAM simulation

**Fig. 3:**
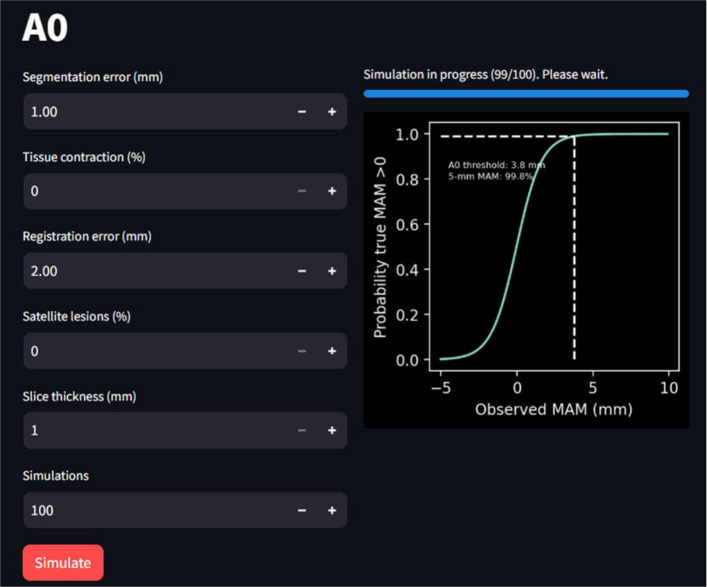
Web application for simulating the influence of measurement errors on A0 for a given software. MAM, minimum ablative margin

The simulations and A0 definition were integrated into a web application to estimate the A0 threshold for a given software and to estimate the probability of achieving a > 0-mm true MAM if a > 5-mm MAM threshold was observed. The web application was implemented in Python and Streamlit (version 1.29.0) and is available at https://acs-accuracy.streamlit.app/. After entering the technical parameters of the software and anticipated biological effects, the simulations were performed, and the thresholds were estimated (Fig 3).

### Statistical analysis

The A0 cutoff for each permutation of the simulation parameters was computed on a high-performance computing cluster. The sigmoid functions were plotted using ggpubr and ggplot2 [27, 28]. A contour plot was further used to plot the A0 threshold for segmentation and registration errors between 1 and 5 mm.

The analysis was performed in R (version 4.4.0) and RStudio (version 2024.04.0). The complete code for the statistical analysis is available in the Github code repository (https://github.com/ipa/acs-accuracy).

## Results

A total of 10,000 simulations for each of the 1500 parameter permutations were performed (*N* = 15,000,000 total simulations).

### Registration error

**Fig. 4:**
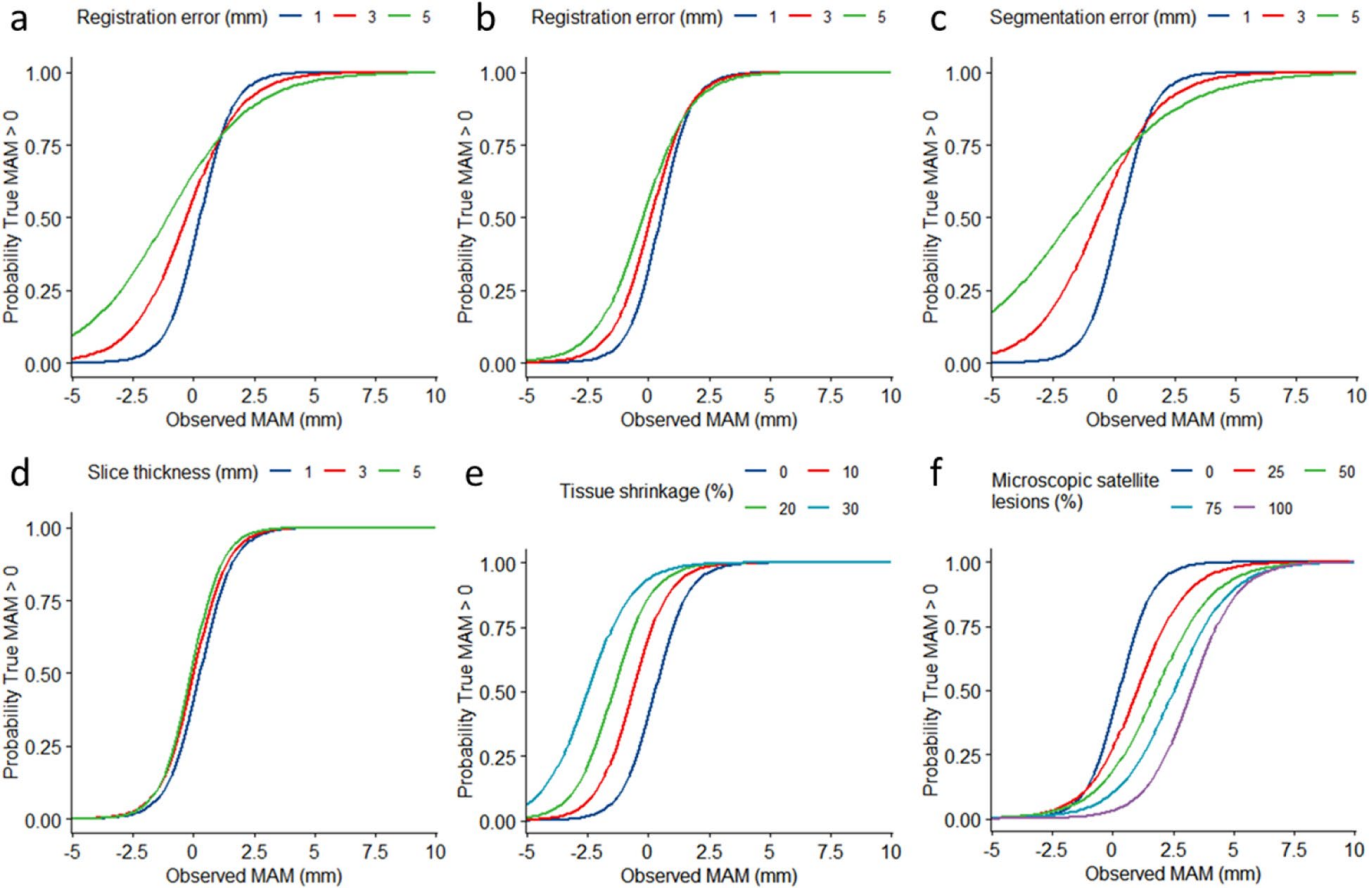
Comparison of how different measurement inaccuracies influence the definition of A0: registration error (**a**), registration error (when excluding registrations with > 3 mm of error) (**b**), segmentation error (**c**), slice thickness (**d**), tissue shrinkage (**e**), and microscopic satellite lesions (**f**). MAM, minimum ablative margin

Higher registration errors led to higher required A0 thresholds, as the A0 threshold needs to incorporate the margin of error of the registration process itself. For registration errors of 1, 3, and 5 mm, the A0 thresholds were 3.4, 4.9, and 7.0 mm, respectively (Fig. 4a). Most studies trying to identify optimal MAM thresholds for A0 exclude cases with inaccurate registrations, commonly using a 3-mm threshold measured by visual inspection at anatomical landmarks [8, 9]. Figure 4b shows the effects of excluding inaccurate registrations for registration errors of 1, 3, and 5 mm and demonstrates that the A0 threshold found with this practice was lower than when all registration measurements were used. Overall, the difference in A0 thresholds increased with increasing registration errors.

### Segmentation error

A greater segmentation error led to a higher A0 threshold, and the segmentation error more greatly influenced the A0 threshold than the registration error. For segmentation errors of 1, 3, and 5 mm, the A0 thresholds were 3.4, 5.2, and 8.4 mm, respectively (Fig. 4c).

### Slice thickness

With larger slice thickness, the A0 threshold marginally decreased (≤ 0.5 mm, which is below the resolution of the simulation and could be attributed to noise in the measurement or simulation). For slice thicknesses of 1, 3, and 5 mm, the A0 thresholds were 3.4, 3.2, and 2.9 mm, respectively (Fig. 4d).

### Tissue shrinkage

Tissue shrinkage during microwave ablation reduced the A0 threshold. The A0 thresholds for 0%, 10%, 20%, and 30% tissue shrinkage were 3.4, 2.8, 2.2, and 1.8 mm, respectively (Fig. 4e).

### Microscopic satellite lesions

The presence of microscopic satellite lesions, which were undetectable on radiologic imaging, increased the A0 threshold, even if not all tumors presented with microscopic satellite lesions. The A0 thresholds for 0%, 25%, 50%, 75%, and 100% probability of the presence of satellite lesions were 3.4, 5.8, 7.4, 7.9, and 7.7 mm, respectively (Fig. 4f).

### Validity of 5-mm A0 threshold

**Fig. 5:**
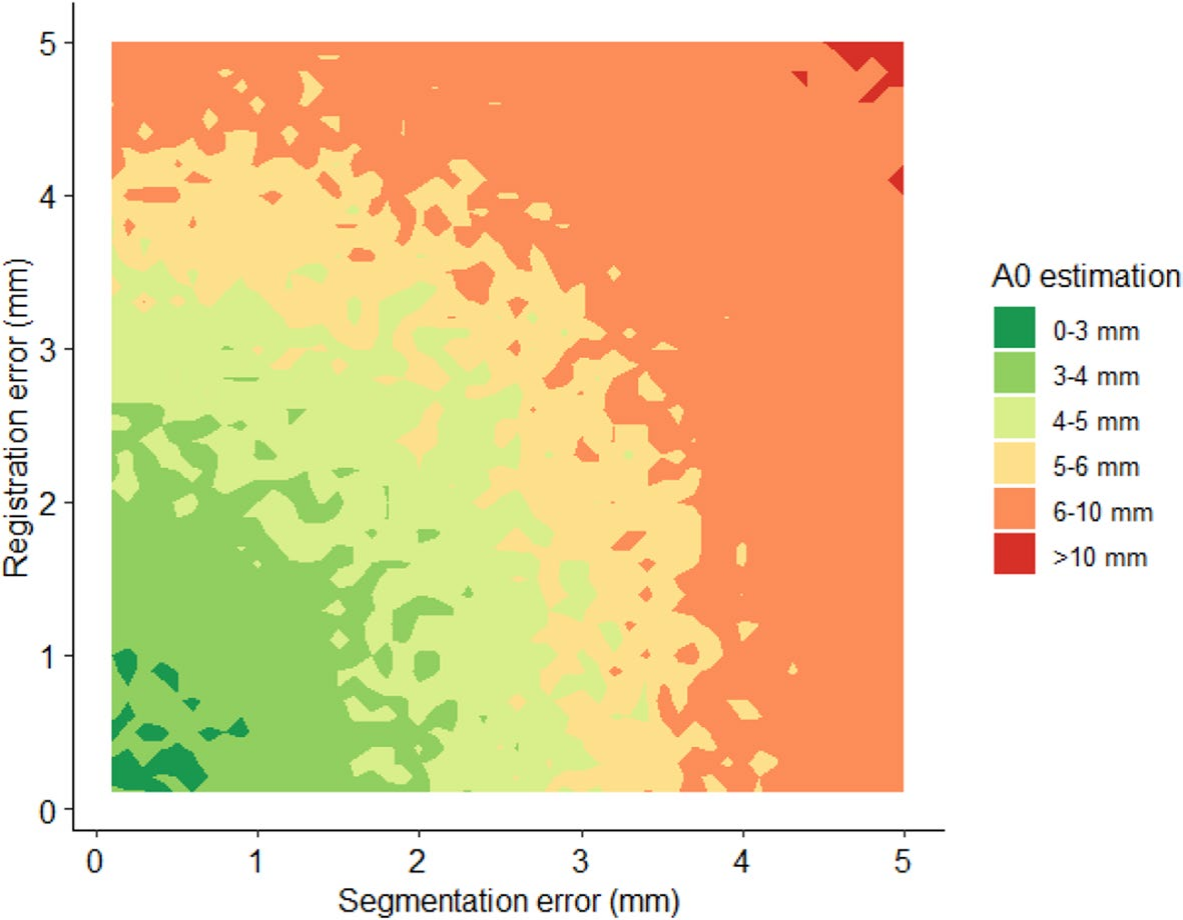
Contour plot of the A0 threshold depending on registration and segmentation errors

Figure 5 shows the A0 threshold depending on segmentation and registration errors—the 2 most important sources of error. Segmentation and registration errors between 1 and 3 mm resulted in A0 thresholds of ≤ 5 mm. Errors larger than 3 mm resulted in larger A0 thresholds, and thus the commonly used 5-mm threshold would be unreliable in those cases.

## Discussion

In this study, we developed a simulation framework to determine how measurement inaccuracies impact A0 thresholds based on cross-sectional imaging. We integrated this simulation into an interactive web application designed to help clinicians estimate the appropriate A0 threshold—and assess the validity of a 5-mm MAM threshold—for their specific AC software. We found that tumor and ablation zone segmentation and registration accuracy greatly affect the definition of A0, whereas slice thickness has a negligible effect. Thus, significant effort should be invested in those related algorithms, especially in registration methods, as errors in these parameters can often not be corrected during the procedure. While the results suggest thicker slices lead to better results, this is likely noise from the simulations as the difference is below the image resolution. Based on our simulations, to achieve an A0 threshold of ≤ 5 mm, an AC system must have a segmentation error of ≤ 3 mm and a registration error of ≤ 3 mm. However, if both errors were exactly 3 mm, a 5-mm A0 threshold would be unreliable. Further, because the exclusion of inaccurate registrations affects the A0 definition, the number of excluded cases needs to be disclosed in scientific studies.

The current literature on the factors influencing A0 thresholds is limited, as each study uses one specific software implementation to process all images. Thus, comparing different software implementations with sufficient sample sizes (N > 1000) to capture the large variation in tumor sizes, margins, and measurement errors is impractical. Furthermore, biological effects like microscopic satellite lesions are undepictable on clinically available cross-sectional imaging and therefore only known probabilistically from histological studies. Therefore, for rare tumor types, which are more prone to have microscopic satellite lesions (e.g., intrahepatic cholangiocarcinoma), it is impossible to collect the required sample size to study the effects of microscopic satellite lesions on A0 thresholds. The same challenge occurs with tissue shrinkage, which is known only from ex vivo studies and cannot be individually measured. The results of our study showed that these biological effects have a large influence on the definition of A0. Thus, in clinical practice, assumptions about these effects should be carefully considered when deciding on MAM requirements.

This study has limitations. First, as a simulation study, certain influences might not have been modeled. For example, the real tissue shrinkage during thermal ablation is still unknown, but in this study, we assumed it to be uniform and of specific magnitudes. Tumors and ablation zones were also modeled as spheres and ellipsoids which represent a simplification of these irregularly shaped structures. Second, we ignored the interactions between the sources of error, which vary depending on the algorithm. For example, a biomechanical deformable registration algorithm would be less accurate if the underlying segmentation algorithms is inaccurate. Similarly, an intensity-based registration might be affected by lower signal-to-noise ratios owing to smaller slice thickness. Differences in slice thickness and other image reconstruction parameters (e.g., mAs and kV), which were not modeled, could also influence segmentation and registration accuracies. Third, segmentation and registration errors were modeled as following a normal distribution with a mean of 0 but varying standard deviation, assuming no systematic errors. Although this might be the case for certain software packages, we assumed that such systematic errors would be corrected during the development of those algorithms, which may not be true for all software packages. We also did not incorporate differences in registration types such rigid vs. deformable as these are heavily implementation dependent.

## Conclusions

In conclusion, this study showed how measurement errors and biological effects influence A0 cutoffs. Our simulation represents an important tool for estimating the required MAM depending on technical errors of the measurement method and biological effects. This in silico model could be used a priori to verify technical performance requirements. In addition, we provided a benchmark for the minimum accuracy necessary for AC software.

## Data Availability

Code and data are available on GitHub: https://github.com/ipa/acs-accuracy.
